# Motivated with joy or anxiety: Does approach-avoidance goal framing elicit differential reward-network activation in the brain?

**DOI:** 10.3758/s13415-024-01154-3

**Published:** 2024-01-30

**Authors:** Michiko Sakaki, Kou Murayama, Keise Izuma, Ryuta Aoki, Yukihito Yomogita, Ayaka Sugiura, Nishad Singhi, Madoka Matsumoto, Kenji Matsumoto

**Affiliations:** 1 Hector Research Institute of Education Sciences and Psychology, University of Tübingen, Tübingen, Germany; 2 School of Psychology and Clinical Language Sciences, University of Reading, Reading, UK; 3 Research Institute, Kochi University of Technology, Kochi, Japan; 4 School of Psychology, University of Southampton, Southampton, UK; 5 School of Economics & Management, Kochi University of Technology, Kochi, Japan; 6 Research Institute for Future Design, Kochi University of Technology, Kochi, Japan; 7 Graduate School of Humanities, Tokyo Metropolitan University, Tokyo, Japan; 8 Araya Inc, Tokyo, Japan; 9 Brain Science Institute, Tamagawa University, Machida, Japan; 10 Graduate Training Centre of Neuroscience, University of Tübingen, Tübingen, Germany; 11 Kyoto University, Kyoto, Japan

**Keywords:** Approach/avoidance, Achievement goal, Behavioral inhibition, Behavioral activation, Dopamine, Goal framing, Neuroimaging

## Abstract

Psychological research on human motivation repeatedly observed that approach goals (i.e., goals to attain success) increase task enjoyment and intrinsic motivation more strongly than avoidance goals (i.e., goals to avoid failure). The present study sought to address how the reward network in the brain—including the striatum and ventromedial prefrontal cortex—is involved when individuals engage in the same task with a focus on approach or avoidance goals. Participants reported stronger positive emotions when they focused on approach goals, but stronger anxiety and disappointment when they focused on avoidance goals. The fMRI analyses revealed that the reward network in the brain showed similar levels of activity to cues predictive of approach and avoidance goals. In contrast, the two goal states were associated with different patterns of activity in the visual cortex, hippocampus, and cerebellum during success and failure outcomes. Representation similarity analysis further revealed shared and different representations within the striatum and vmPFC between the approach and avoidance goal states, suggesting both the similarity and uniqueness of the mechanisms behind the two goal states. In addition, the distinct patterns of activation in the striatum were associated with distinct subjective experiences participants reported between the approach and the avoidance conditions. These results suggest the importance of examining the pattern of striatal activity in understanding the mechanisms behind different motivational states in humans.

**Supplementary Information:**

The online version contains supplementary material available at 10.3758/s13415-024-01154-3.

Decades of research on human motivation addressed the reasons behind inter- and intra- individual differences in the intensity and frequency of various behaviors (Braver et al., 2014). These studies pointed out that even if two individuals work on the same task with the intent to produce the same outcome, their emotional experiences, task engagement and behavior (which we shall call “motivational states”) can be different depending on how they perceive the characteristics of the task (Ryan et al., 1996). The literature also identified various factors that affect people’s motivational states (Kruglanski, 2017; Murayama, 2022; Reeve, 2018). One of the most investigated and robustly observed factors is the difference of approach versus avoidance goals (Carver et al., 2000; Elliot, 1999). Approach goals are defined as goals to obtain desirable outcomes (“My goal is to gain X”), whereas avoidance goals are defined as goals to avoid the loss of desirable outcomes (“My goal is to avoid losing X”).

In public communication as well as psychological research on human motivation, the salience of approach and avoidance goals often is manipulated by changing the frame of messages or instructions—whether they focus on the benefits of adopting certain behaviors (*an approach frame*; e.g., “Regular exercise helps you control your weight and blood pressure”) or the costs of not adopting certain behaviors (*an avoidance frame*; e.g., “Without regular exercise, you are more likely to become obese and suffer from high blood pressure”; Bertolotti & Catellani, 2014). Using such methods, previous laboratory studies repeatedly observed that even if individuals face the same task, their subjective experiences and strategies are different depending on whether their goal is framed as approach or avoidance. In general, approach goals increase task enjoyment and intrinsic motivation, whereas avoidance goals increase anxiety and other negative emotions (Elliot & Harackiewicz, 1996; Gee et al., 2018; Higgins et al., 1997). Similar results were observed in field studies by using questionnaires to assess individual differences in the strength of approach versus avoidance goals (for a meta-analysis, see Van Yperen et al., 2014).

How are these different motivational states represented in the brain? Previous neuroscience research often posited that the reward network in the brain, including the striatum, the substantia nigra, the ventral tegmental area (VTA), and ventromedial prefrontal cortex (vmPFC), is critical for intrinsic motivation (Di Domenico & Ryan, 2017; Lee & Reeve, 2017; Murayama et al., 2010; Reeve & Lee, 2019b)—the motivation for the pleasure of the task itself (Deci & Ryan, 1985). Given that approach goals typically increase positive emotional states (including intrinsic motivation), these studies thus suggest that the reward network shows greater activity when individuals pursue approach goals than avoidance goals. A similar prediction has been suggested by a personality theory based on the behavioral approach system (BAS) and the behavioral inhibition system (BIS) (Gray, 1982, 1990). The BIS/BAS are closely related to approach/avoidance goals; those who have higher BAS scores are more sensitive to gains and rewards and therefore have higher approach goals, whereas those who have higher BIS scores are more sensitive to losses and punishments and therefore have higher avoidance goals (Carver & White, 1994). According to Gray, the reward network plays key role in BAS than BIS. In line with his notion, past studies found that those with higher BAS (but not BIS) showed greater activation of the striatum towards monetary rewards (Carter et al., 2009; Costumero et al., 2016; Hahn et al., 2009; Simon et al., 2010) or food rewards (Beaver et al., 2006). Recent research further revealed that stronger BAS scores (but not BIS) were associated with stronger functional connectivity within the reward network (Adrián-Ventura et al., 2019).

However, other research suggested that the reward network reflects more general motivational engagement independent from positively valenced subjective feelings (Carter et al., 2009; Reeve & Lee, 2019a; Sakaki et al., 2023). In fact, a recent meta-analysis revealed that the striatum, a key region in the reward network, is activated not only when individuals expect monetary rewards but also when they expect monetary losses (Oldham et al., 2018; White et al., 2021). These notions thus suggest that the reward network may show similar levels of activity across approach and avoidance goals. This prediction is supported by previous neuroimaging studies that used an experimental manipulation on approach versus avoidance goals. For example, Schlund et al. (2011) used a response learning task, where participants earned money when they made a correct response (the approach frame) and lost money when they made incorrect response (the avoidance frame). They found that the reward network showed similar levels of activities to cues irrespective of the goal frame. Belayachi et al. (2015) also revealed that the frontoparietal areas showed similar levels of activities when participants completed a working memory task under approach and avoidance goals. While this study is not necessarily about the reward network, their results suggest that the two goals are represented similarly in the brain.

In addition, other studies showed more complex pictures that were not consistent either of the aforementioned views. For example, Scult et al. (2017) examined the effects of individual differences in prevention vs. promotion focus (a concept closely related to avoidance vs. approach goals) on brain activities during a monetary reward task (i.e., Monetary Incentive Delay task). They found that higher promotion focus was associated with *smaller* activity in the ventral striatum to reward than loss cues. Other studies suggest that the approach versus avoidance goals are associated with the striatum in different hemispheres (Aberg et al., 2015; Eddington et al., 2007; Spielberg et al., 2011, 2012). Thus, it is still inconclusive with regards to the role of the reward network in the approach versus avoidance goals.

One challenge for these past studies is that most of them used monetary rewards as part of the tasks of approach (i.e., to earn money) and avoidance (i.e., not to lose money) manipulations. Given that the reward network is activated by the presence of monetary rewards (Adcock et al., 2006) as well as the pleasure of tasks (Murayama et al., 2010), it is not clear from these studies whether the observed activity of the reward network is due to gains/loss of monetary rewards versus changes in intrinsic motivation (inherent pleasure of tasks). In fact, Sakaki et al. (2023) showed that when participants were engaged in a game-like, intrinsically enjoyable task, the activation pattern in the reward network was different, depending on whether participants worked on the task for monetary incentives or just for the enjoyment of the task itself. This is because when people are incentivized for monetary rewards, their motivation to gain rewards and to enjoy the task often would be in conflict, making the interpretation difficult. In addition, previous neuroimaging studies on approach/avoidance goals do not include measures on subjective motivational states during tasks (Belayachi et al., 2015; Oldham et al., 2018; Schlund et al., 2011; Scult et al., 2017; White et al., 2021). Therefore, it has been unclear how the reward network in the brain is similarly or differently involved in positive and negative subjective motivational states induced by approach versus avoidance goals.

In the present study, we conducted a neuroimaging experiment to examine whether and how the reward network in the brain is involved in approach versus avoidance goals by using a game-like, intrinsically motivating task. Participants completed a stopwatch task (a task that was proven to be intrinsically engaging for adults; Murayama et al., 2010, 2013; Sakaki et al., 2023) while being scanned in an MRI scanner. They were instructed that they could win or lose points during the stopwatch task depending on their performance, and their overall goal was to earn the total point of zero or larger than zero at the end of the experiments. They were also told that the points they would earn would have nothing to do with their monetary rewards. The goal frame was manipulated—such that participants completed the task under an approach goal frame in some blocks but under an avoidance goal frame in other blocks. In the approach blocks, participants gained points by succeeding in the task (i.e., approach goal framing); in the avoidance blocks, participants lost points by failing the task (i.e., avoidance goal framing). Note that both approach and avoidance blocks were functionally equivalent to achieve the overall goal. For example, even if participants fail all the trials in the approach condition, if they succeed in all the trials in the avoidance condition, the total points at the end of the experiment would be zero, thus achieving the overall goal. Similarly, participants should be able to achieve the overall goal by succeeding in all the trials in the approach condition—in this case, even if participants fail all the trials in the avoidance condition, the total points at the end of the experiment would be zero. Thus, both the approach and avoidance blocks had the equal “importance” in terms of achieving the overall goal of the task.

We assessed participants’ subjective motivational states after each block (in the scanner) as well as at end of the study session. Building on previous findings (Elliot & Harackiewicz, 1996), we expected that the two types of blocks would cause different motivational states in participants, with the approach blocks producing positive intrinsic motivational states (e.g., more enjoyment) while the avoidance blocks arousing negative motivational states (e.g., more anxiety). We also expected that participants would develop implicit associations between these different motivational states and tasks over time (Greenwald et al., 2003).

As brain regions relevant to different motivational states between the two goal frames, we focused on the reward network in the brain; including the striatum, the substantia nigra, VTA, and vmPFC. These brain regions have been robustly activated by the task cue as well as success outcomes in previous studies with the same task even without any extrinsic incentives (Murayama et al., 2010, 2013; Sakaki et al., 2023; Takeda et al., 2017). Activation in the reward network during the stopwatch task was interpreted as the manifestation of intrinsic enjoyment for the task (Murayama et al., 2010). Therefore, they were a natural choice for our regions-of-interest (ROIs).

In our functional MRI analyses, we examined brain activity to a cue which indicated the start of the task (i.e., when participants anticipated an upcoming task) and brain activity towards outcomes (i.e., when participants found out whether they were successful). These two phases correspond to “expected” versus “actual experience of” reward value respectively (Schultz, 2002). More specifically, during the cue phase (Fig. 1), participants were informed of the goal frame (i.e., approach or avoidance) of an upcoming trial as well as how many points they would subsequently win if they succeeded in the approach condition or how many points they would lose if they failed in the avoidance condition. Building on the previous studies that approach goals make people focus on expected positive outcomes (Elliot & Harackiewicz, 1996), we expected that participants would anticipate more pleasant emotions in the approach condition than in the avoidance condition. If the reward-network activation reflects positive motivational states (e.g., intrinsic motivation), these brain regions should show enhanced activity to the task cue in the approach condition but not to the task cue in the avoidance condition.

**Fig. 1:**
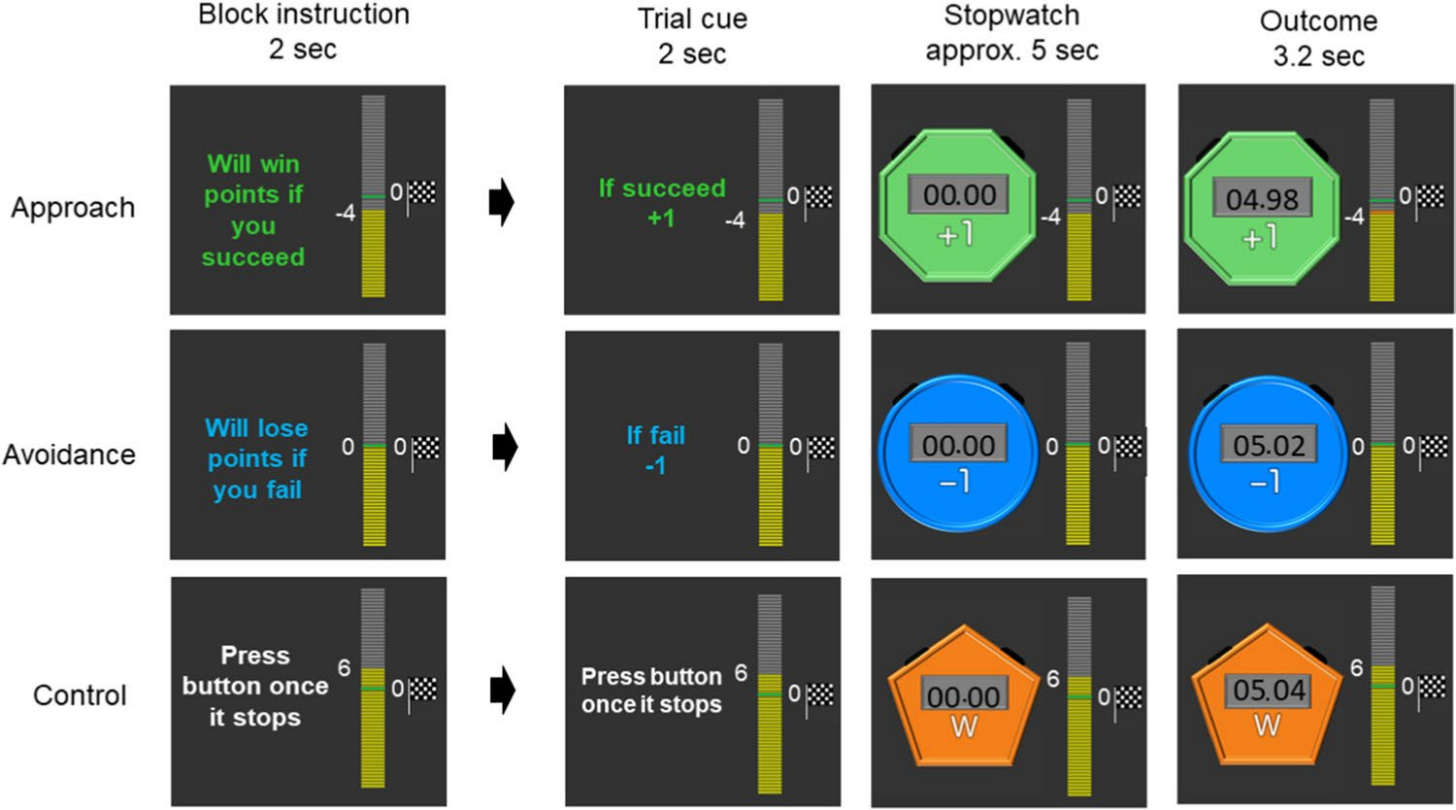
Task in the approach, avoidance, and control conditions

During the outcome phase (Fig. 1), participants found out whether they were successful or not in each particular trial. Previous psychological research has suggested that success outcomes are perceived more positively under the approach goal frame than the avoidance goal frame (Idson et al., 2000). Thus, if the reward network activity reflects intrinsic enjoyment, these brain regions also would be activated more strongly by the success feedback than the failure feedback in the approach condition but not in the avoidance condition. In contrast, if the reward-network activation reflects general motivational saliency or engagement dissociated from positive or negative motivational states, the reward network should show a similar level of activation to the cue and the success outcome in the approach and avoidance condition.

We also analyzed whether the goal frames affect functional connectivity of the reward network and representations within the reward network. Even when the overall level of activity is similar across the two goal frames, the functional connectivity of the reward network may be different across the two goal states. Given the nucleus accumbens (NAc)’s dense projections with various brain regions (Haber & Knutson, 2010), we examined whether functional connectivity of the NAc is different depending on the goal states. Likewise, even when the overall level of activity is similar, the two goal states may be supported by different patterns of activation within the reward network. Previous research pointed out that a representational similarity analysis is effective to address this issue and to provide insights into whether different tasks are supported by shared or distinctive neural mechanisms (Freund et al., 2021; Levorsen et al., 2021; Peelen & Downing, 2007). To this end, we also applied a representational similarity analysis and examined if the pattern of activity in the reward network is similar or different when participants expect the approach and avoidance goals.

## Methods

### Participants

Twenty participants (*M*_age_ = 20.55, standard deviation [SD] = 1.43; 9 males and 11 females) took part in the study. Written consent from participants was obtained based on the protocol approved by the Ethics Committee of the Tamagawa University.

### Design and behavioral procedure

The overall design and procedure is depicted in Fig. 1. Participants completed a game-like task (the stopwatch task; Murayama et al., 2010, 2013; Sakaki et al., 2023) under approach and avoidance goal framing. For each trial, participants were presented with a stopwatch that automatically started; they were asked to presss a button to stop the stopwatch within 50 ms of the 5-s time point. If participants stopped the stopwatch within this time window, the trial was deemed a success. In contrast, if participants failed to stop the stopwatch within this time window, the trial was deemed as a failure. To experimentally manipulate the success/failure outcome (without being affected by participants’ performance), participants were shown random numbers on the display of the stopwatch after 3 s; thus, they could not accurately calibrate the correct timing. In some blocks, participants completed the task with a focus on approach goals, where they earned points when they succeeded but did not lose any points when they failed. In other blocks, participants completed the same task with a focus on avoidance goals, where they lost points when they failed but did not earn points when they succeeded.

Each block started with a short instruction indicating the type of the upcoming block (approach = “Will win points if you succeed”; avoidance = “Will lose points if you fail”). Participants needed to press a button within 5 s to indicate that they understood the nature of the upcoming block; when participants did not press a button within 5 s, they were told that they needed to respond quickly and shown the same instruction again. This block instruction was followed by a jittered ISI (between 3 to 7 s) and experimental trials. Each trial started with a cue for 2 s. The cue indicated one of the three different points (3, 1, and 0). In the approach blocks, participants were told that they would earn the presented amount of points when they were successful in the trial, but they would not lose any points if they failed in the trial. In the avoidance blocks, participants were told that they would lose the presented amount of points when they failed in the trial, but they would not earn any points when they were successful. The cue was followed by a jittered ISI (between 3 to 7 s), which was replaced by a stopwatch that automatically started. Participants needed to press a button to stop the stopwatch within the time frame described earlier. Once participants pressed the button, the stopwatch stopped and participants found out whether they were sucessful or not. The outcome was shown for 3.2 s, followed by a jittered ITI (3–7 s) and the next trial. When participants failed to press a button within 6 s after the stoptwatch started, they were told to press the button sooner and a new trial started. The three different point conditions were included to increase the unpredictability of the task and thereby encourage participants to pay attention to the cue of each trial.

In the remaining blocks, participants completed a watch-stop game (i.e., the control condition). Each trial in this control task started with a cue (Fig. 1). Participants then passively viewed a stopwatch that automatically started and stopped; they pressed a button after the stopwatch stopped. In this control condition, they did not earn/lose any points. When participants pressed the button too quickly in the watch-stop task (i.e., before the stopwatch stopped), they were instructed to press the button only after the stopwatch stopped and the new trial started.

The experiment included 117 trials in total. These 117 trials were divided into three runs, each of which included five to eight blocks. This resulted in the total of 19 blocks with different lengths (each block included three to nine trials). There were eight blocks for the approach condition (48 trials), eight blocks for the avoidance condition (48 trials), and three blocks for the control condition (21 trials). For both approach and avoidance blocks, we presented the three different points (3, 1, 0) evenly (i.e., 16 trials for each point) but pseudorandomly; this means that participants were not able to predict the amount of points in the next trial. A stopwatch was colored differently across three conditions (blue, green, or orange); the color assignment was counterbalanced across participants.

The amount of points participants earned accumulated across blocks and runs; the total points they earned were indicated by a progress bar shown throughout the task. Before the task, participants were instructed that their overall goal of the experiment was to earn the total of zero or larger than zero at the end of the fMRI session. Participants were always reminded of this goal by a small flag that was displayed next to the progress bar (Fig. 1).

To ensure that our results were not confounded with task performance itself (or individual differences in the progress toward the goal), unbeknownst to participants, success versus failure outcome was experimentally controlled. Participants’ success or failure outcome was predetermined for every trial, and participants were presented with the predetermined outcome regardless of their actual performance. Because we occluded the stopwatch after 3 s, participants did not realize our manipulation (no participants indicated their doubt after the experiment). We prepared four different sequences of success-failure outcomes and randomly assigned one of them to each participant. In all sequences, we made sure that participants could not be confident until the last trial about whether they could achieve the overall goal; participants finished the task with a total of zero points for all sequences.

### Behavioral measures

We assessed participants’ motivational states for approach-avoidance blocks in three ways. First, after each block, participants rated the level of enjoyment (“I enjoyed the block”) and anxiety that they felt during the block they had just completed (“I was anxious during the block”) both with a 5-point Likert scale (*not at all* – *extremely*) while in the scanner. The enjoyment and anxiety scores were averaged across all the blocks for each condition respectively and used as an index of on-task measures of motivational states.

Second, after they exit the scanner, participants filled out a retrospective self-report questionnaire about emotional/motivational states they experienced during the approach and avoidance blocks. The questionnaire included two items for each of the following aspects with a 7-point Likert scale (*not at all true* – *very true*): enjoyment (e.g., “I enjoyed the approach/avoidance blocks”), anxiety (e.g., “I had anxiety during the approach/avoidance blocks”), disappointment (e.g., “I was disappointed when I had to work on the approach/avoidance blocks”), engagement (e.g., “I felt engaged for the approach/avoidance blocks”), and excitement (e.g., “I was excited with the approach/avoidance blocks”). Ratings given to the two items were averaged for each condition for each participant and were used as a measure that reflects retrospective evaluations of motivational states. The questionnaire also included items on emotions participants felt after the success and failure outcomes in each condition. Specifically, participants were asked to indicate the extent to which they felt happiness and relief when they were successful in the approach trials and avoidance trials respectively. They also indicated the extent to which they felt disappointed and anxious when they were not successful in the approach trials and avoidance trials. For both types of questions, we used a 7-point Likert scale (1: “not at all true” – 7: “very true”). The questions were asked for the points of 3, 1, and 0 separately and we averaged the scores for the 3 and 1 points as we focused on these trials in the fMRI data analysis (see below).

Finally, to assess participants’ implicit attitude toward the approach and avoidance blocks, we asked participants to complete an Implicit Association Test (ITA) at the end of the experiment (Greenwald et al., 2003). Participants were presented with positive/negative words as well as two pictures of stopwatches, one being used in the approach block (“approach stopwatch”) and the other being used in the avoidance block (“avoidance stopwatch”). Across seven IAT blocks (for details see Greenwald et al., 2003), participants were asked to press one of the two buttons in response to each of the presented stimuli. In two blocks, positive words and the approach stopwatch were assigned to the same key, while negative words and the avoidance stopwatch were assigned to the other key. In the other two blocks, positive words and the avoidance stopwatch were assigned to the same key, whereas negative words and the approach stopwatch were assigned to the other key. If participants had developed a positive attitude toward a stopwatch used in the approach condition, they should be able to respond faster and more accurately when the approach stopwatch was assigned to the same key as positive words than negative words. Based on the response time and error rate data, we computed IAT scores as the index of participants’ implicit attitudes (in this paper, higher scores mean that individuals developed more positive implicit attitudes toward the approach stopwatch than the avoidance stopwatch). There are different algorithms proposed to compute the IAT scores, but we report the one recommended by Greenwald et al. (2003), although the results were the same with other computation algorithms.

### Behavioral analysis

Participants’ responses in the self-report questions after each block and at the end of the experiment were compared between the approach and the avoidance conditions. We also analyzed IAT. We applied the false discovery rate (FDR) correction to control the type-I error rate.

### MRI data acquisition

Data were acquired by using a 3-Tesla Siemens Trio Tim MRI scanner. Functional scans were obtained during three runs in the main task with echo T2*-weighted echo-planar images (EPI; repetition time = 2500 ms; echo time = 25 ms; flip angle = 90°; slice thickness = 3 mm; the number of slices = 42; acquisition matrix = 64 × 64). In addition, for each participant, a whole-brain, high-resolution T1-weighted structural scan was acquired (repetition time = 2000 ms, echo time = 1.98, acquisition matrix = 256 x 256). For functional data, we discarded the first two images before data processing and statistical analysis.

### FMRI preprocessing

Data preprocessing was performed by using FMRIB's Software Library (FSL ver 6.0.5; www.fmrib.ox.ac.uk/fsl), including skull stripping of structural images with BET, motion correction with MCFLIRT, smoothing with full-width half-maximum 5 mm, and high-pass temporal filtering (Gaussian-weighted least-squares straight line fitting, with sigma = 100 s). We next performed MELODIC Independent Component Analysis (ICA; Beckmann & Smith, 2004); artifact components were then removed with FSL’s fMRIB's ICA-based Xnoiseifier (FIX; Salimi-Khorshidi et al., 2014). Registration was performed with FLIRT; each functional image was registered to the participant’s high-resolution brain-extracted structural image by using a 7 degree of freedom (dof) transformation, and each participant’s high-resolution structural image was registered to the standard Montreal Neurological Institute (MNI) 2-mm brain using a 12 dof transformation.

### FMRI analysis

Image analysis was performed using FSL FEAT (fMRI Expert Analysis Tool, https://fsl.fmrib.ox.ac.uk/fsl/fslwiki/FSL). For each run for each participant, the BOLD responses were modeled with a general linear model (GLM) with the following effects: 1) trial cues separately modeled for each point (0, 1, or 3) for each goal orientation condition (approach or avoidance), 2) success feedback for each point for each goal orientation condition, 3) failure feedback for each point for each goal orientation condition, and 4) trial cues for the stop-watch condition. The model also included the block instruction for each condition, motion parameters, the rating phases, missed block instruction (where participants failed to respond) and missed trials (where participants pressed a button too quickly or too slowly) as regressors of no interest.

To address our research questions, we defined our contrasts of interest for cue phases as well as outcome phases. For all contrasts described below, we did not include approach/avoidance trials with 0 points to make the interpretation straightforward. Regarding the cue phases, we focused on the following contrasts: a) the approach cue > the avoidance cue; b) the avoidance cue > the approach cue; c) the approach cue > the control cue; and d) the avoidance cue > the control cue. Because we were also interested in the responses to success and failure outcomes across the two goal conditions, we included the following contrasts for outcomes: a) the approach success > the approach failure; b) the approach failure > the approach success, c) the avoidance success > the avoidance failure; d) the avoidance failure > the avoidance success; e) the approach success > the avoidance success; f) the approach failure > the avoidance failure; g) the avoidance success > the approach success; and h) the avoidance failure > the approach failure. The outputs from the first-level analysis were merged across runs for each participant by using a fixed-effects analysis in FSL’s FEAT. The outputs from these fixed-effects analyses were further entered into a random effects analysis using FSL’s FEAT FLAME 1. Both in the region-of-interest (ROI) analysis and the whole brain analysis, we employed cluster-based corrections for multiple comparisons with Gaussian random field theory (*Z* = 3.1; cluster significance: *p* = .05 corrected).

#### Region-of-interest (ROI) analysis

We first conducted a small-volume correction analysis, focusing on the following regions implicated in reward processing: the striatum, the substantia nigra, VTA, and vmPFC. The substantia nigra and VTA masks were provided by Jessica Mollick, drawn based on Eapen et al. (2011). The bilateral striatum mask was based on the Harvard-Oxford Subcortical Probability Atlas with the probability of 20% for the NAc, the putamen, and the caudate. We *a priori* decided a relatively lenient threshold (i.e., 20 %) given that a larger ROI mask leads to a more stringent threshold for significance in our analysis. The vmPFC mask was obtained based on the meta-analysis from de la Vega et al. (2016). These masks were then combined and used as a mask for our small-volume correction analysis. To visualize the activity patterns across the conditions, we used FSL’s featquery to extract beta values from significant voxels observed in the ROI analysis.

#### Whole-brain analysis

We also performed a whole-brain analysis to examine how the goal frames and the outcome affect brain activity in other parts of the brain. The same set of contrasts used in the ROI analyses were also used in the whole brain analysis.

### Functional connectivity analysis

Condition-dependent changes in functional connectivity of the NAc were examined using the generalized psychophysiological interaction (gPPI) approach (McLaren et al., 2012) using FSL. While the ROI and whole brain analysis described earlier focused on event-related analysis, our gPPI analysis focused on changes in functional connectivity across the approach versus avoidance blocks. Therefore, we did not model a cue for each trial and outcome for each trial as separate regressors. Instead, we included two block regressors (one for the approach block and the other for the avoidance block), a time series of a seed region, the interaction between the time series and the approach condition, and the interaction between the time series and the avoidance condition in FSL’s FEAT. We also included the onset of the cue for the control block, missed block instructions (where participants failed to respond), and the rating phase as additional regressors. The connectivity map from each run was concatenated across three runs for each participant with a fixed-effects analysis in FSL’s FEAT. The participant-level maps were then used in the random-effect analysis done by FSL’s FEAT FLAME 1. The seed regions included voxels in the left and right (separately) NAc that showed significant effects during the ROI analysis. As in our ROI analysis, NAc was defined by the Harvard-Oxford Subcortical Probability Atlas with the probability of 20%. We used FSL’s command-line utility “fslmeants” to extract time series of the seed regions from the functional data. As in the whole brain analysis described earlier, results were considered significant at Z > 3.1 and cluster-corrected *p* < .05.

### Representational similarity analysis

We also applied representational similarity analysis (RSA) (Freund et al., 2021) to examine whether the pattern of activity within the reward network is similar when participants expected the stop-watch task in the approach goal condition, the stop-watch task in the avoidance goal condition, and the control task. Pre-processing of functional imaging data was re-run without spatial smoothing and ICA denoising, and the functional data for each participant were not transformed to MNI space (Lee et al., 2018; Weaverdyck et al., 2020). We modeled a cue for each point (0/1/3) for each condition separately for each participant; success/failure outcomes, motion parameters, rating, and block cues were also included as regressors of no interest. Using FSL FEAT, we obtained beta values for each voxel for each regressor, resulting in a voxel-wise pattern of beta values for each cue type. As in the other analyses, we focused on approach and avoidance trials with 1 or 3 points. We also focused on the striatum and vmPFC; these regions were defined as in the ROI analysis described above. Using PyMVPA (http://www.pymvpa.org/) (Hanke et al., 2009), we computed Pearson correlations across different runs, points and conditions based on the voxel-wise activation pattern. The correlation coefficients were transformed by Fischer’s z-transformation before statistical analyses to compare them.

To examine whether the activation patterns were more similar for two conditions from the same goal frame than two conditions from different goal frames, we compared the similarity in the approach-approach pairs (i.e., similarity between the approach condition with 1 point and the approach condition with 3 points), avoidance-avoidance pairs (i.e., similarity between the avoidance condition with 1 point and the avoidance condition with 3 points), and approach-avoidance pairs (e.g., similarity between the approach condition and the avoidance condition). We also examined whether the pattern similarity was higher for two stop-watch conditions with different goal frames (i.e., approach-avoidance pairs) than the stopwatch-control pairs (i.e., the similarity between the approach condition and control condition or between the avoidance condition and control condition).

As an exploratory analysis, we next explored whether distinct representations in the striatum and vmPFC were associated with different subjective experiences across the approach and avoidance goal conditions. For each participant for each ROI (striatum and vmPFC), we computed a pattern similarity index by dividing the similarity in representations between the approach and avoidance pairs by the similarity between the two same goal pairs (i.e., approach-approach and avoidance-avoidance); higher values represent higher similarity in the voxel activation patterns between the approach and avoidance condition. For each self-report measure, we obtained a difference score between the two goal conditions for each participant (e.g., on-task enjoyment for the approach condition minus on-task enjoyment for the avoidance condition); we applied Principal Component Analysis (PCA) to all the difference scores from self-reports and used the first component as an index for the general difference in subjective experiences between the two goal conditions (see Table S3 for loadings). Given the presence of potential outliers, we used Spearman’s rank correlation and examined if this general difference in subjective experiences was significantly associated with the pattern similarity indices for the striatum and the vmPFC.

## Results

### Behavioral results

**Fig. 2:**
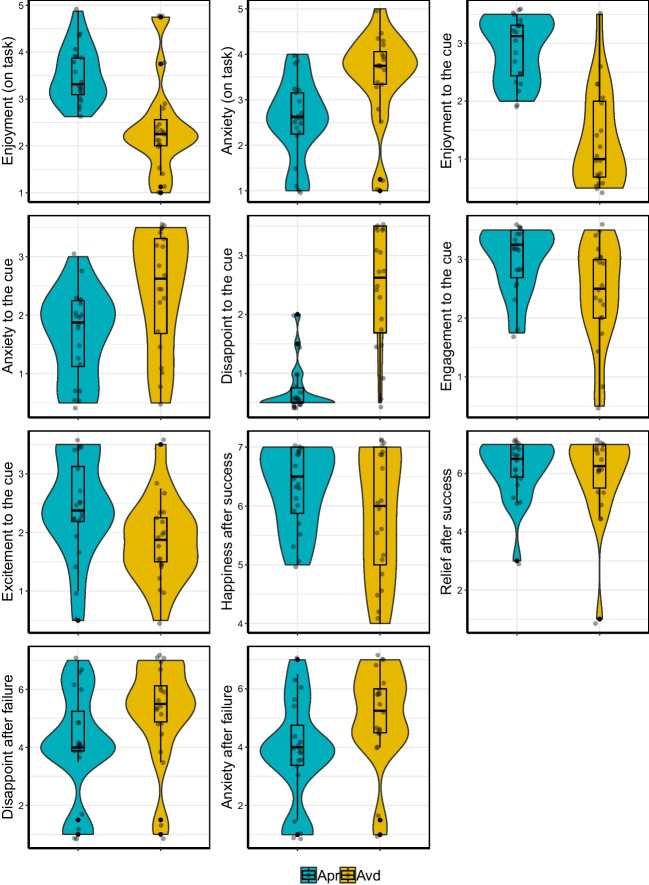
Behavioral results for the approach (“Apr”) and the avoidance (“Avd”) conditions

In this section, we adjusted *p*-values based on FDR. Participants overwhelmingly demonstrated positive emotions and stronger motivation for the approach blocks than for the avoidance blocks (Fig. 2). This was evident in the on-task rating, where participants reported stronger enjoyment (*M*apr = 3.52, SD = 0.61; *M*avd = 2.41, SD = 1.01), *t*(19) = 5.07, *p* < .01, dz = 1.13, and weaker anxiety after the approach than the avoidance blocks (*M*apr = 2.57, SD = 0.89; *M*avd = 3.52, SD = 1.00), *t*(19) = 4.45, *p* < .01, dz = −0.99. Likewise, in the post-scanning retrospective ratings, participants reported stronger enjoyment (*M*apr = 5.80, SD = 1.09; *M*avd = 2.65, SD = 1.69), *t*(19) = 7.36, *p* < .01, dz = 1.65, less anxiety (*M*apr = 3.33, SD = 1.52; *M*avd = 4.88, SD = 2.05), *t*(19) = −3.29, *p* < .01, dz = −0.74, less disappointment (*M*apr = 1.40, SD = 0.80; *M*avd = 4.85, SD = 2.03), *t*(19) = −6.44, *p* < .01, dz = −1.44, more engagement (*M*apr = 5.95, SD = 1.15; *M*avd = 4.90, SD = 1.72), *t*(19) = 2.59, *p* < .05, dz = 0.58, and more excitement (*M*apr = 4.88, SD = 1.69; *M*avd = 3.70, SD = 1.40), *t*(19) = 3.64, *p* < .01, dz = 0.81, to the cue for the approach condition than the avoidance condition.

When asked about emotions towards success and failure feedback, participants reported stronger happiness towards success in the approach trials than the avoidance trials (*M*apr = 6.30, SD = 0.70; *M*avd = 5.82, SD = 1.09), *t*(19) = 2.37, *p* < .05, dz = 0.53. Participants also reported stronger disappointment (*M*apr = 4.10, SD = 1.85; *M*avd = 5.20, SD = 1.67), *t*(19) = 4.00, *p* < .01, dz = −0.89, and stronger anxiety (*M*apr = 3.85, SD = 1.76; *M*avd = 5.08, SD = 1.66), *t*(19) = 3.89, *p* < .01, dz = −0.87, toward failures in the avoidance trials than the approach trials. There was no significant difference in the feelings of relief between the two conditions (*M*apr = 6.12, SD = 1.02; *M*avd = 5.95, SD = 1.44), *t*(19) = 1.10, dz = 0.25. In addition, the IAT scores reveal that participants developed more positive attitude toward the approach blocks than the avoidance blocks (*M* = 95.03, SD = 113.79); *t*(19) = 3.73, *p* < .01, dz = 0.84. As shown in the effect size metric, most of the effects were relatively large (except for relief), indicating the large differences between the blocks in terms of subjective feelings.

### ROI analysis

#### Activity to cue

**Fig. 3:**
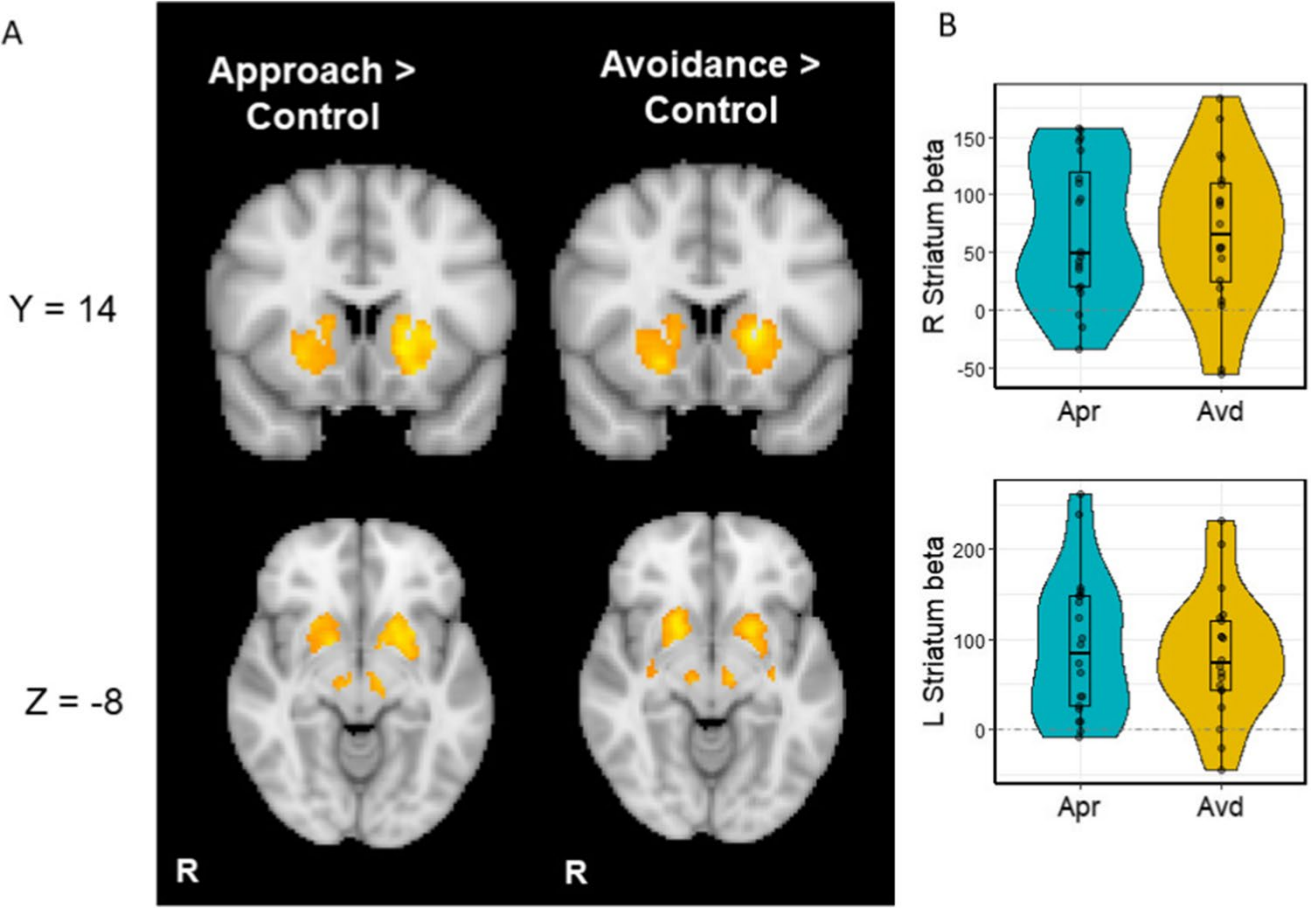
(A) Striatum activity to cues predictive of the approach and avoidance goals respectively relative to the control condition. (B) Beta values extracted from the significant striatum cluster. Apr = approach, Avd = avoidance. Dashed lines represent activity in the control condition

**Fig. 4:**
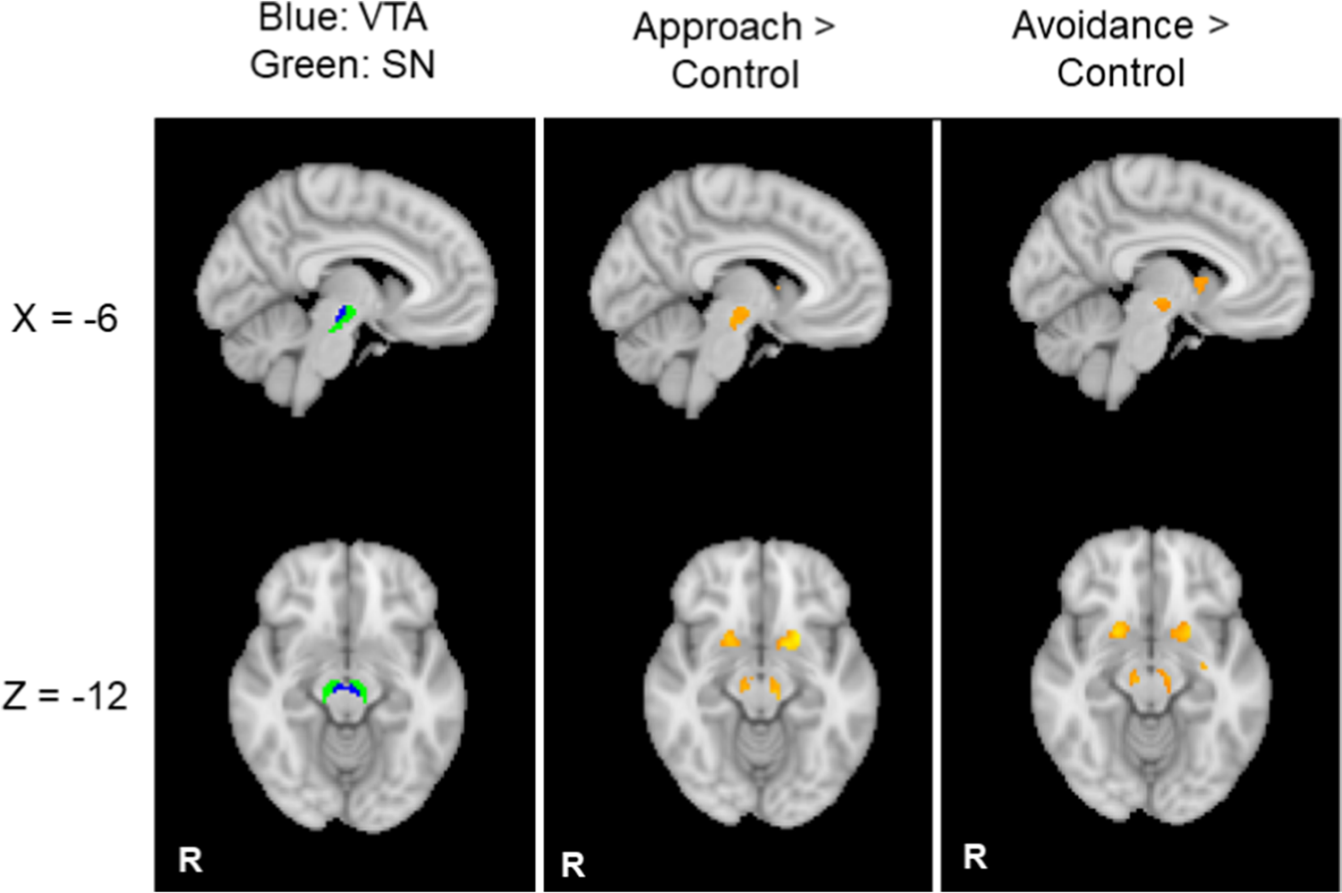
The ventral tegmental area (VTA) and substantia nigra (SN) showed higher levels of activity to cues predictive of the approach and the avoidance goals relative to the control condition

Relative to the control cues (i.e., watch-stop cues), the cues for the approach and the avoidance conditions induced stronger activation in the bilateral striatum, including the caudate, nucleus accumbens (NAc) and putamen (Fig. 3A-B; Table 1). We also found significant clusters around the brainstem, including the VTA and SN, which showed greater activity to the cues for the approach and the avoidance conditions relative to the cue for the control condition (Fig. 4; Table 1). There were no significant differences between the approach vs. avoidance conditions. These results suggest that, unlike the behavioral results, the levels of activations in the reward network were not significantly affected by the approach-avoidance goal manipulation.

**Table 1:** Significant clusters and local maxima in each cluster during cue presentations in the ROI analysis

Contrast	Voxels	Area	Z stat	H	x	y	z
*Approach > Control*							
	1063	Putamen	5.22	L	-16	12	-2
		Putamen	5.13	L	-22	10	-12
		Putamen	5.04	L	-26	2	2
		Putamen	4.88	L	-22	6	6
		Putamen	4.86	L	-16	6	-12
		Putamen	4.7	L	-24	-4	8
	814	Putamen	4.71	R	24	6	6
		Putamen	4.61	R	16	4	-8
		Putamen	4.52	R	24	16	-4
		Putamen	4.42	R	24	-8	10
		Putamen	4.16	R	20	10	-12
		Putamen	4.1	R	34	0	2
	84	Brainstem	4.23	L	-8	-22	-12
		Brainstem	4.12	L	-10	-16	-8
		Brainstem	3.84	L	-4	-16	-16
	82	Brainstem	3.96	R	8	-20	-10
		Brainstem	3.94	R	8	-22	-16
		Brainstem	3.91	R	6	-14	-6
		Brainstem	3.91	R	6	-14	-14
		Thalamus	3.89	R	10	-18	-6
*Avoidance > Control*							
	1077	Caudate	5.53	L	-18	10	0
		Caudate	5.2	L	-20	6	6
		Caudate	5.18	L	-24	0	-2
		Caudate	5.12	L	-24	2	2
		Putamen	4.95	L	-20	8	-8
		Caudate	4.79	L	-22	-6	6
	858	Caudate	5.19	R	16	6	-6
		Putamen	5.11	R	20	10	-8
		Caudate	4.84	R	22	2	2
		Caudate	4.32	R	22	-2	12
		Caudate	4.27	R	10	0	10
		Putamen	4.21	R	24	-6	12
	53	Brainstem	4.36	L	-10	-18	-8
	48	Brainstem	4.24	R	10	-18	-10
*Approach > Avoidance*							
		NS					
*Avoidance > Approach*							
		NS					

Corresponding brain region was identified by the Harvard Oxford Cortical/Subcortical atlas. *NS* no significant results

#### Activity to outcome

**Fig. 5:**
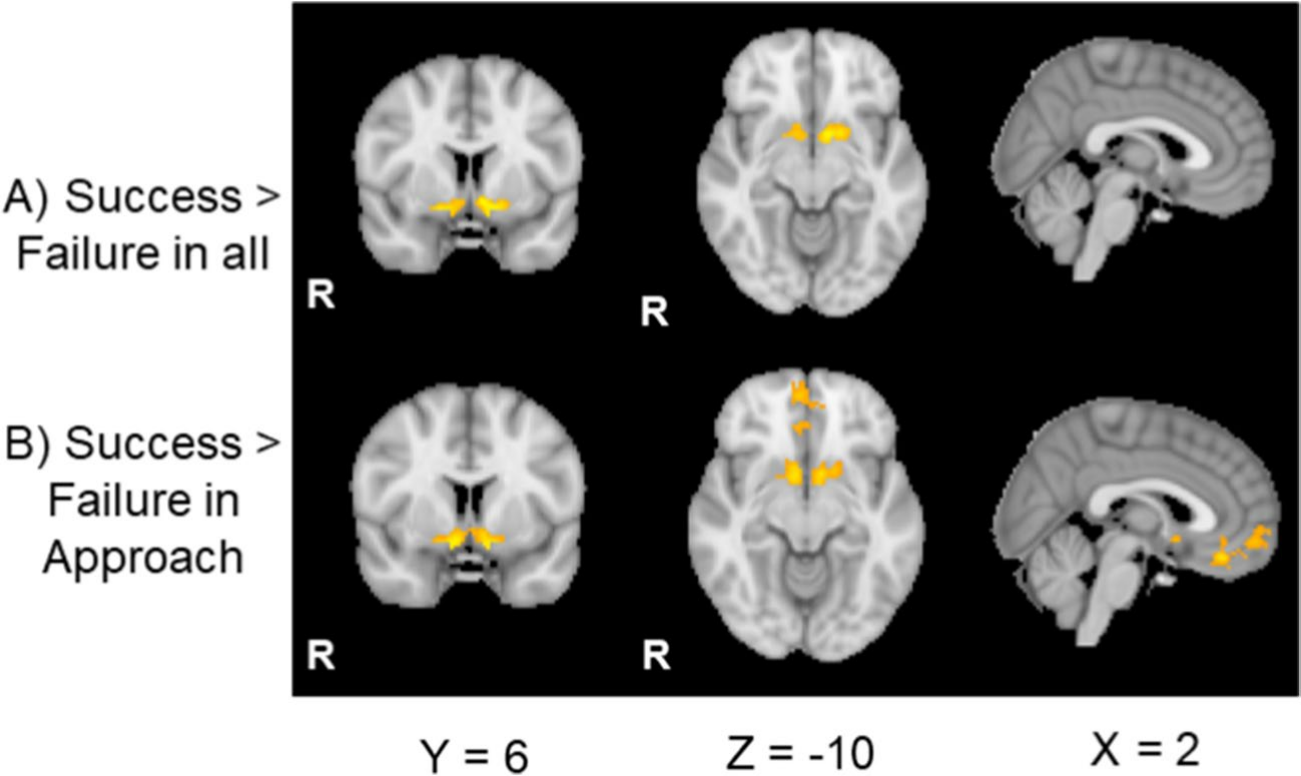
The activity in the striatum and vmPFC to success than failure outcomes in the ROI analysis

During the outcome phase, we found greater activity in the bilateral NAc to success relative to failure outcomes (Fig. 5A; Table 2), replicating previous findings (Murayama et al., 2013). When examining the approach and avoidance conditions separately, the significant NAc activity after success outcomes was observed only in the approach condition (Fig. 5B; Table 2). In the approach condition, vmPFC also showed a significant activity to success than failure outcomes. In contrast, in the avoidance blocks, there were no significant differences between success versus failure outcomes (Table 2). However, we did not find a significant interaction between the goal type (approach vs. avoidance) and outcome (success vs. failure) in the ROI analysis (Table 2). Thus, there is no strong evidence that the effects of success versus outcomes were different in our ROIs between the two goal conditions (Fig. S1).

**Table 2:** Significant cluster during success than failure outcome presentations in the ROI analysis

Contrast	Voxels	Area	Z stat	H	x	y	z
*Success > Failure*							
	151	Caudate	4.42	L	-8	8	-8
	79	Putamen	4.11	R	16	6	-12
		Nucleus accumbens	4.05	R	10	4	-14
		Nucleus accumbens	3.72	R	8	12	-8
		Frontal orbital cortex	3.41	R	16	12	-12
Approach Success > Approach Failure							
	304	Nucleus accumbens	4.83	R	10	4	-14
		Nucleus accumbens	4.66	L	-10	4	-14
		Putamen	3.78	R	18	6	-12
		Putamen	3.58	L	-20	14	-12
		Subcallosal cortex	3.5	L	-2	8	-6
	188	Frontal medial cortex	4.06	R	6	54	-8
		Frontal medial cortex	3.81	L	-4	50	-8
		Frontal pole	3.7	L	-2	60	0
		Frontal medial cortex	3.68	L	-2	54	-6
		Frontal pole	3.47	R	4	64	-4
	134	Frontal medial cortex	4.35	R	2	36	-18
		Paracingulate gyrus	3.52	R	6	36	-8
		Paracingulate gyrus	3.28	R	0	38	-10
		Frontal medial cortex	3.27	R	2	50	-18
Avoidance Success > Avoidance Failure							
		NS					
Approach Success > Avoidance Success							
		NS					
Avoidance Failure > Avoidance Success							
		NS					
Avoidance Failure > Approach Failure							
		NS					
*[Approach Success - Approach Failure] > [Avoidance Success - Avoidance failure]*							
		NS					
*[Avoidance Success - Avoidance Failure] > [Approach Success - Approach Failure]*							
		NS					

Corresponding brain region was identified by the Harvard Oxford Cortical/Subcortical atlas. *NS* no significant results

### Functional connectivity analysis

We next used the gPPI analysis to explore how the functional connectivity of the striatum is different between the approach and the avoidance conditions. The seeds were the left and right NAc that showed significant activity to the success outcomes than the failure outcomes in the approach condition. However, we did not find any significant differences in the functional connectivity with the right/left NAc across the conditions.

### Representational similarity analysis

**Fig. 6:**
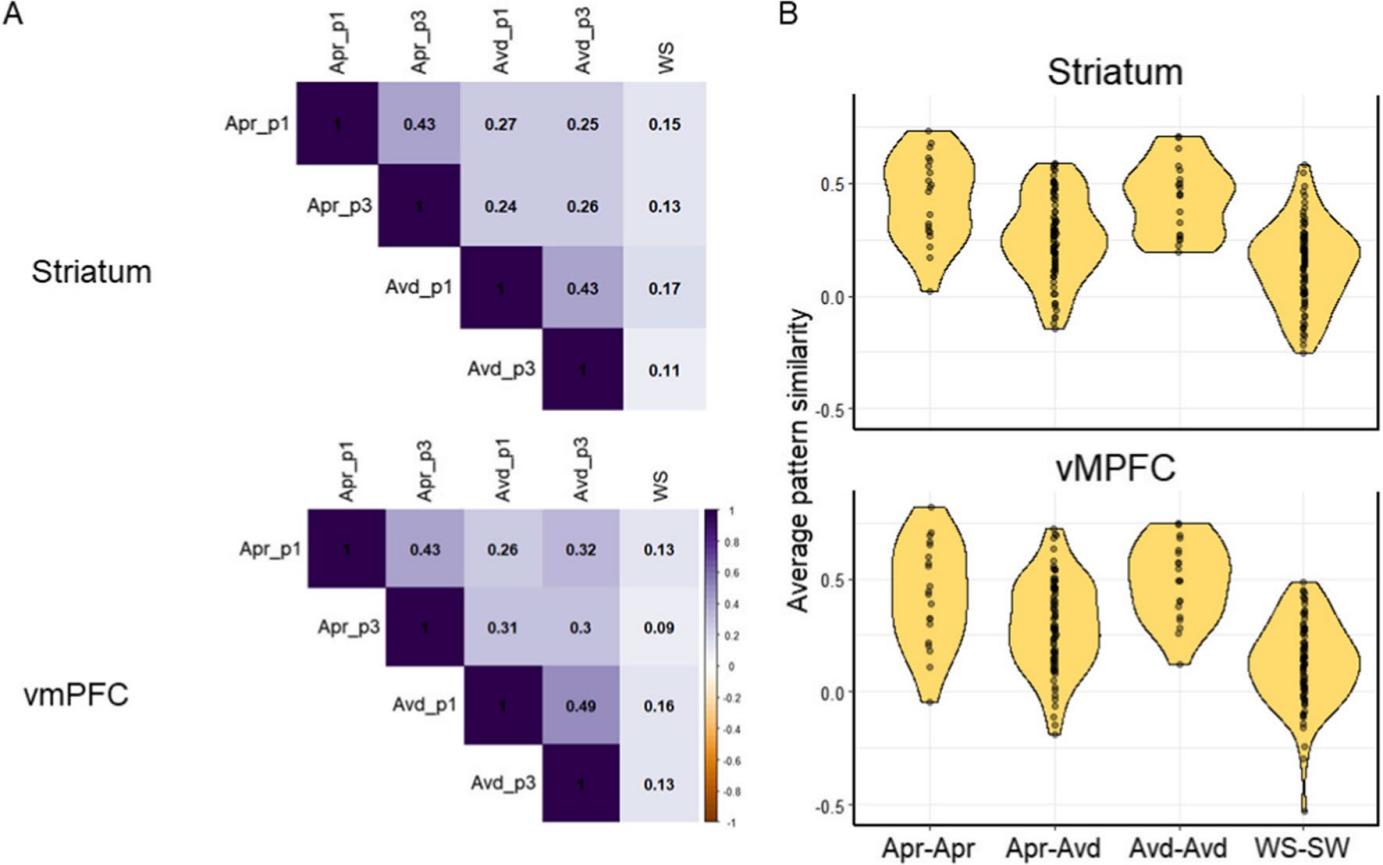
Results from the representational similarity analysis for the approach (“Apr"), avoidance (“Avd”), and control conditions (“WS”). (**A**) The averaged similarity matrix for the striatum and vmPFC across all cues. p1: point 1 trials, p3: point 3 trials. (**B**) The pattern similarity was the highest for the same goal pairs (Apr-Apr: approach-approach pairs and Avd-Avd: avoidance-avoidance pairs), followed by pairs of stop-watch tasks with different goal frames (Apr-Avd; approach-avoidance pairs). The Apr-Avd pairs still had higher similarity than when they were paired with control tasks (WS-SW: watch-stop – stopwatch pairs)

Using RSA, we examined the similarity in the pattern of activity in voxels within the striatum and vmPFC across cues for the approach condition, the avoidance condition, and the control condition (Fig. 6A). The activity pattern in the striatum and vmPFC was significantly correlated across all pairs (*ps* < .05 FDR corrected). However, as shown in Fig. 6A, the similarity was higher for pairs that included two stop-watch tasks (irrespective of the goal frames) than for pairs that included a stop-watch task and a watch-stop/control task. In addition, cues for the stop-watch task showed higher similarity when they were paired with the other cue for the same goal frame than when they were paired with other cues for the different goal frame.

**Fig. 7:**
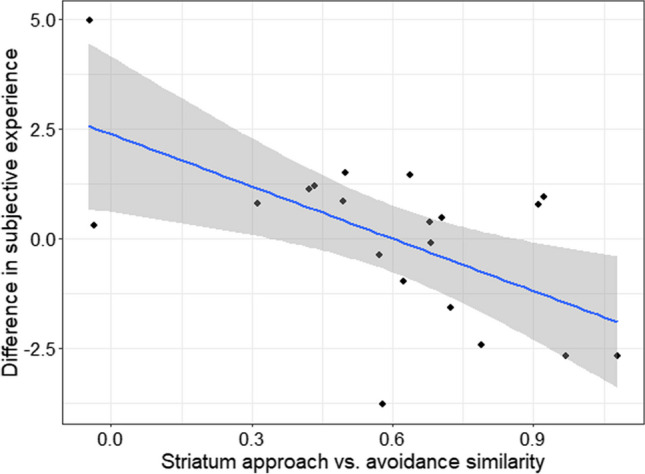
Lower similarity in the striatum representations between the approach and avoidance condition was associated with higher differences in subjective experience between the two conditions

In fact, a 2 (region: striatum vs. vmPFC) x 4 (pair types: approach-approach, avoidance-avoidance, approach-avoidance, and approach/avoidance-control) ANOVA on the similarity measures resulted in a significant effect of pair types, *F*(3, 76) = 28.33, *p* < .001, η_G_ = .35, without any other significant effects (*p*s > .20; Fig. 6B). Follow-up analyses revealed that the activation pattern was more similar for the same goal-type pairs (approach-approach: *M*_striatum_ = .43, SD = .16; *M*_vmPFC_ = .43, SD = .17; avoidance-avoidance: *M*_striatum_ = .43, SD = .19; *M*_vmPFC_ = .49, SD = .23) than the approach-avoidance pairs (*M*_striatum_ = .26, SD = .16; *M*_vmPFC_ = .30, SD = .17), *ts*(19) = 3.62, 6.26, d = 0.92, 1.27, *ps* < .05 (Tukey). However, the approach-avoidance pairs still had higher similarity than the control-stopwatch pairs (*M*_striatum_ = .14, SD = .18; *M*_vmPFC_ = .13, SD = .19), *t*(19) = 3.72, d = 1.20, *p* < .01 (Tukey). These results suggest that while there are unique representations for the approach and avoidance goals in the striatum and vmPFC, the two goals also share patterns of activity that are significantly different from the control/watch-stop condition. An exploratory analysis further revealed that the striatum’s pattern similarity index was significantly associated with distinct subjective experiences between the two goal conditions, Spearman’s rho = −.53, *p* = .017. This means that those who had more *dissimilar* representations in the striatum between the approach and avoidance goal conditions experienced more distinct subjective experiences across the two conditions (Fig. 7). The pattern similarity index of vmPFC was not significantly associated with subjective experiences (*p* = .67).

### Whole-brain analysis

Our analysis so far focused on the striatum, VTA/SN and vmPFC. To address whether the two goal states were differently associated with other brain regions, apart from the reward network in the brain, we also performed a whole brain analysis.

#### Activity to cue

A whole-brain analysis on brain activity during the cue phase confirmed results from our ROI analyses that both the approach and avoidance cues induced greater activity in the striatum and the VTA/SN than the control cues (Fig. S2; Table S1). Both the approach and avoidance cues induced greater activity in the cingulate gyrus, supramarginal gyrus, the middle frontal gyrus, the inferior frontal gyrus, and the occipital pole relative to the control cue. There were no significant differences between approach and avoidance cues.

#### Activity to outcome

**Fig. 8:**
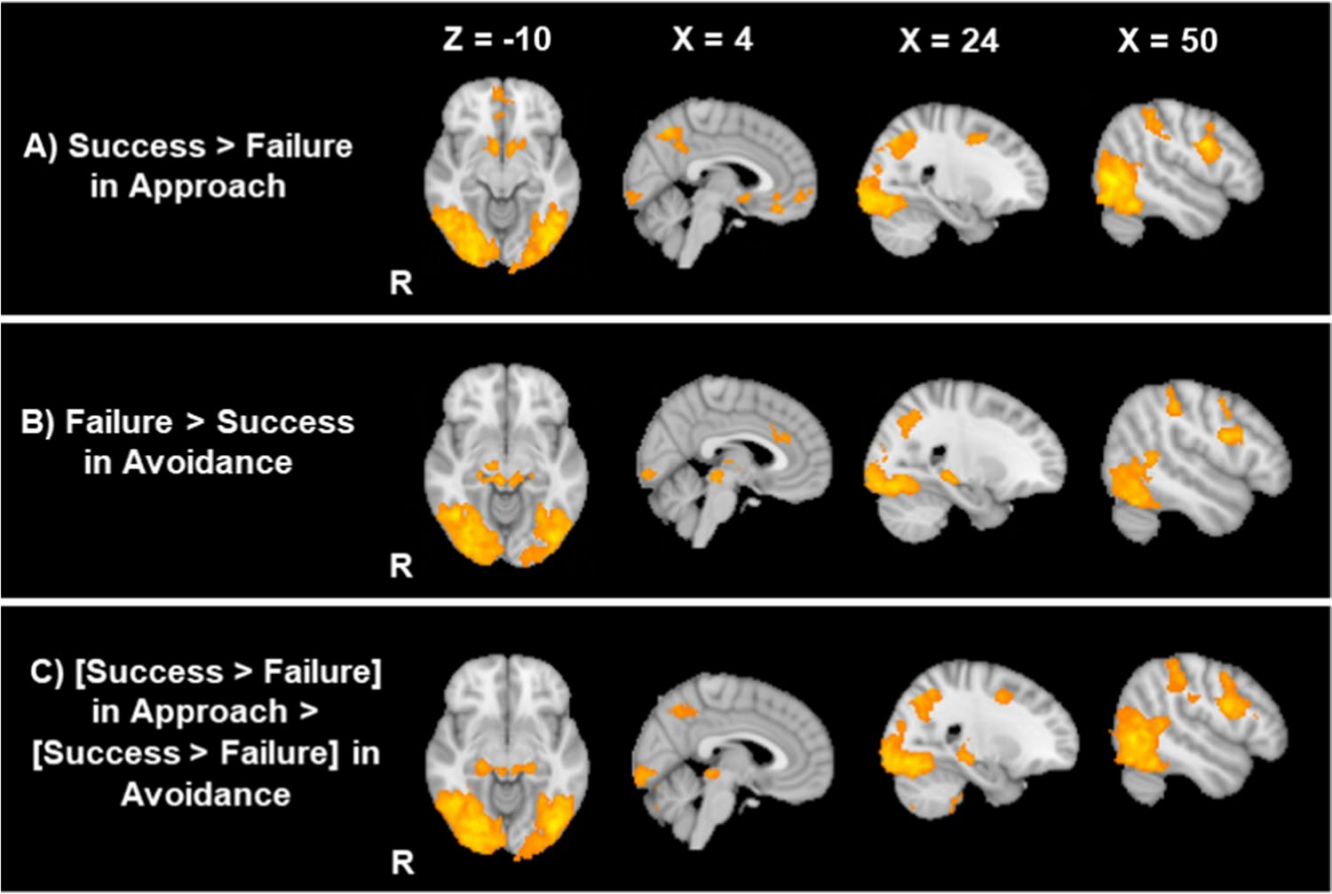
Whole-brain results for the success and failure outcomes for each goal frame condition

A whole-brain analysis during the outcome phases also confirmed the results from the ROI analysis that NAc and vmPFC showed greater activity to success than failure outcomes in the approach block (Table 3; Fig. 8A). Beyond these regions, in the approach condition, success outcomes induced greater activity in the bilateral lateral occipital gyri, the bilateral precentral gyrus/the middle frontal gyrus as well as the cerebellum than did failure outcomes (Fig. S3). In contrast, we did not find any regions that showed greater activity to failure than success outcomes in the approach condition.

**Table 3:** Significant clusters and local maxima during the outcome phase in whole-brain analysis

Contrast	Voxels	Area	Z stat	H	x	y	z
*Approach Success > Approach Failure*							
	10471	Lateral occipital cortex	6.05	R	50	-66	-2
		Lateral occipital cortex	5.84	R	48	-68	-16
		Occipital fusiform gyrus	5.7	R	36	-70	-16
		Temporal occipital fusiform cortex	5.64	R	40	-58	-16
		Occipital fusiform gyrus	5.61	R	32	-74	-14
		Temporal occipital fusiform cortex	5.6	R	44	-56	-22
	8897	Lateral occipital cortex	6.04	L	-42	-68	-14
		Lateral occipital cortex	5.99	L	-24	-66	46
		Lateral occipital cortex	5.97	L	-46	-66	-6
		Superior parietal lobule	5.94	L	-30	-56	48
		Lateral occipital cortex	5.84	L	-24	-60	50
		Lateral occipital cortex	5.79	L	-44	-76	-12
	978	Precentral gyrus	5.56	R	50	6	28
		Precentral gyrus	4.19	R	54	6	18
		Superior frontal gyrus	4.12	R	24	10	46
		Superior frontal gyrus	4.04	R	22	0	44
		Precentral gyrus	3.99	R	34	-4	42
		Precentral gyrus	3.88	R	44	2	42
	664	Nucleus accumbens	4.83	R	10	4	-14
		Nucleus accumbens	4.66	L	-10	4	-14
		Amygdala	4.39	L	-12	-2	-16
		Frontal orbital cortex	4.12	R	18	8	-16
		Frontal orbital cortex	3.92	L	-20	14	-14
		Subcallosal cortex	3.5	L	-2	8	-6
	652	Precentral gyrus	4.78	L	-46	4	30
		Precentral gyrus	4.47	L	-44	0	36
		Precentral gyrus	4.08	L	-42	2	20
		Precentral gyrus	4.08	L	-52	6	36
		Inferior frontal gyrus	3.78	L	-38	16	20
		Inferior frontal gyrus	3.47	L	-52	22	26
	198	Frontal medial cortex	4.06	R	6	54	-8
		Frontal medial cortex	3.81	L	-4	50	-8
		Frontal pole	3.7	L	-2	60	0
		Frontal medial cortex	3.68	L	-2	54	-6
		Frontal pole	3.47	R	4	64	-4
	149	Frontal medial cortex	4.35	R	2	36	-18
		Frontal medial cortex	3.59	R	10	38	-16
		Paracingulate gyrus	3.52	R	6	36	-8
		Paracingulate gyrus	3.28	L	0	38	-10
		Frontal medial cortex	3.27	R	2	50	-18
	145	Cerebellum	3.89	L	-12	-74	-50
		Cerebellum	3.75	L	-20	-72	-52
		Cerebellum	3.73	L	-6	-82	-42
		Cerebellum	3.57	L	-10	-78	-46
	117	Inferior frontal gyrus	3.78	L	-50	34	16
		Frontal pole	3.72	L	-48	38	10
		Inferior frontal gyrus	3.62	L	-46	26	12
		Frontal pole	3.24	L	-38	42	8
*Avoidance Success > Avoidance Failure*							
		NS					
*Approach Failure > Approach Success*							
		NS					
*Avoidance Failure > Avoidance Success*							
	7458	Occipital fusiform gyrus	5.92	R	34	-66	-18
		Occipital fusiform gyrus	5.83	R	34	-70	-14
		Temporal occipital fusiform cortex	5.79	R	42	-60	-18
		Temporal occipital fusiform cortex	5.7	R	44	-56	-16
		Occipital fusiform gyrus	5.65	R	32	-80	-14
		Temporal occipital fusiform cortex	5.59	R	44	-58	-12
	4255	Temporal occipital fusiform cortex	5.53	L	-32	-56	-16
		Lateral occipital cortex	5.45	L	-40	-80	-10
		Occipital pole	5.41	L	-32	-92	-18
		Temporal occipital fusiform cortex	5.29	L	-34	-60	-20
		Lateral occipital cortex	5.25	L	-44	-74	-8
		Occipital fusiform gyrus	5.2	L	-36	-80	-16
	1176	Hippocampus	4.7	R	20	-30	-6
		Brainstem	4.69	R	6	-30	-10
		Thalamus	4.66	R	12	-20	10
		Thalamus	4.57	R	12	-16	6
		Hippocampus	4.37	R	24	-26	-8
		Thalamus	4.25	L	-12	-30	-4
	573	Inferior frontal gyrus	4.96	R	44	8	24
		Precentral gyrus	4.36	R	42	8	32
		Precentral gyrus	4.29	R	44	0	42
		Precentral gyrus	3.9	R	50	2	48
		Precentral gyrus	3.62	R	48	6	40
	151	Cingulate gyrus	4.09	L	0	22	30
		Cingulate gyrus	3.72	R	4	30	24
		Paracingulate gyrus	3.28	R	6	38	32
		Paracingulate gyrus	3.18	R	8	38	36
	134	Precentral gyrus	4.67	L	-50	0	32
		Precentral gyrus	4.3	L	-52	2	38
*[Avoidance Failure - Avoidance Success] > [Approach Failure - Approach Success]*							
	20435	Occipital fusiform gyrus	6.42	R	36	-72	-16
		Lateral occipital cortex	6.31	R	48	-66	-2
		Occipital fusiform gyrus	6.27	R	32	-80	-14
		Occipital fusiform gyrus	6.27	R	32	-76	-12
		Lateral occipital cortex	6.17	L	-40	-78	-10
		Lateral occipital cortex	6.15	L	-44	-68	-16
	1483	Precentral gyrus	5.33	R	50	4	22
		Inferior frontal gyrus	5.33	R	44	8	24
		Precentral gyrus	4.81	R	42	0	40
		Inferior frontal gyrus	4.56	R	46	12	16
		Precentral gyrus	4.56	R	56	10	32
		Middle frontal gyrus	4.54	R	26	2	46
	890	Hippocampus	5.15	R	20	-30	-6
		Hippocampus	4.81	R	22	-26	-8
		Brainstem	4.6	R	6	-32	-6
		Brainstem	4.36	L	-8	-30	-6
		Hippocampus	4.33	L	-20	-28	-8
		Brainstem	4.18	R	10	-30	-4
	697	Precentral gyrus	4.77	L	-48	4	36
		Inferior frontal gyrus	4.73	L	-48	8	26
		Inferior frontal gyrus	4.71	L	-46	8	22
		Precentral gyrus	4.59	L	-52	-2	38
		Precentral gyrus	4.55	L	-42	2	20
		Precentral gyrus	4.36	L	-40	2	26
	268	Cerebellum	4.33	L	-8	-80	-40
		Cerebellum	4.07	L	-18	-70	-54
		Cerebellum	4.03	L	-22	-70	-56
		Cerebellum	3.6	L	-10	-70	-48
		Cerebellum	3.43	L	-20	-78	-46
	150	Cerebellum	3.88	R	12	-76	-40
		Cerebellum	3.85	R	14	-76	-44
		Cerebellum	3.83	R	6	-78	-34
		Cerebellum	3.82	R	16	-76	-48
		Cerebellum	3.8	R	20	-76	-48
		Cerebellum	3.54	R	14	-82	-46
	109	Cerebellum	3.9	R	22	-38	-46
		Cerebellum	3.81	R	22	-40	-54
		Cerebellum	3.48	R	24	-32	-42
		Cerebellum	3.3	R	32	-40	-40
		Cerebellum	3.27	R	36	-38	-40
*[Avoidance Success - Avoidance Failure] > [Approach Success - Approach Failure]*							
		NS					

Corresponding brain region was identified by the Harvard Oxford Cortical atlas, Harvard Oxford Subcortical atlas as well as the Talairach atlas. *NS* no significant results

In the avoidance condition, the [success > failure] contrast did not reveal any brain regions that showed significant activity (Table 3). However, in the avoidance condition, failure outcomes, relative to success outcomes, evoked stronger activity in the bilateral lateral occipital gyrus, right precentral gyrus/middle frontal gyrus, the right hippocampus, and the right superior part of the brainstem around the superior colliculus (Fig. 8B; Fig. S4). These different effects of success versus failure between the approach and avoidance conditions resulted in significant interactions in bilateral lateral occipital gyrus, bilateral precentral gyrus/middle frontal gyrus, bilateral hippocampus, the cerebellum as well as the bilateral brainstem including the superior colliculus (Fig. 8C). Thus, while the reward network showed similar patterns between approach and avoidance goal conditions, the goal manipulation elicited the opposite pattern for other areas in the brain between success versus failure outcomes.

## General discussion

Despite the functional equivalence of approach and avoidance blocks with regards to their relevance to the overall goal of the task (i.e., to obtain the zero point), participants’ motivational states were markedly different between the two types of blocks. Specifically, the approach goal trials were perceived as more enjoyable, engaging, and exciting, whereas avoidance goal trials were perceived as more anxiety provoking and disappointing. Participants also reported stronger happiness toward success in the approach condition than in the avoidance condition. In contrast, they reported stronger anxiety and disappointment toward failures in the avoidance condition than in the approach condition. Thus, their subjective experiences were substantially different depending on the goal frame.

Nevertheless, the level of activation in the reward network was strikingly similar between the two conditions. For both approach and avoidance goal blocks, the striatum, VTA, and substantia nigra showed significantly higher levels of activation in response to the task cue relative to the control condition, without any significant differences between the approach versus avoidance conditions. Even though the striatum showed significant activity to success outcome only in the approach condition, we did not observe a statistically significant difference between approach and avoidance conditions. In addition, when we investigated the pattern of activity in the reward network using RSA, we found that the activity pattern for the approach condition had higher similarity with the avoidance condition—relative to the watch-stop/control condition.

Previous studies observed increased activation in the reward network during a similar task and interpreted it as the manifestation of positive motivational states, so-called intrinsic motivation (Murayama et al., 2010). In contrast, we observed similar levels of activity in the reward network across the approach and avoidance conditions, even though participants had stronger intrinsic motivation in the former than the latter. Thus, our findings do not corroborate the claim that the reward network simply reflects intrinsic motivation (Di Domenico & Ryan, 2017; Lee & Reeve, 2017; Murayama et al., 2010). Rather, the results suggest that the reward network activation may reflect general motivational engagement, which is free from the valenced emotional feelings during the task. This interpretation is consistent with some of the past observations (Kim, 2013). For example, Sakaki et al. (2023) showed participants 1) a task cue signaling a stopwatch that had a 50% success rate and 2) another cue signaling a stopwatch that was extremely easy for them to succeed (with a 100% success rate). Participants were also given monetary reward after every success. They had more positive emotional feelings about the easy condition than the difficult condition, which made sense as the easy condition enabled them to earn more money. Nevertheless, the task cue with the difficult condition activated the ventral striatum/ventral pallidum more robustly than the task cue for the easy condition. These findings indicate that the striatal activation in response to the task cue does not simply reflect positively valenced feelings; instead, they may reflect motivational engagement (i.e., participants needed to work harder in the difficult condition with stronger engagement relative to the easy condition). Krebs et al. (2011) also conducted an fMRI study, which independently manipulated the reward and task difficulty, and suggested that the reward network (e.g., the midbrain, caudate nucleus) may be related to mental resource recruitment on top of the rewarding value.

A recent, large-scaled study also supports this notion (White et al., 2021); the authors analyzed individual differences in striatal activity during the monetary incentive delay task and found that the striatum was involved not only for reward but also for loss. In addition, they found that the level of the striatal activity was not associated with self-report measures on positive emotions. These results further suggest that the reward network plays an important role for reward as well as general task engagement. Their results are consistent with our findings that different subjective experiences do not necessarily correspond to different levels of activity in the striatum.

Our study also suggests several key differences between the approach and avoidance goals. First, our RSA analyses not only revealed the similarity in the way the approach and avoidance goals are represented in the reward network but also highlighted that the two goal orientations are represented differently within the reward network. Specifically, we found that the pattern of activity within the striatum and vmPFC was more similar in the two stop-watch cues with the same goal orientation (i.e., approach-approach pairs or avoidance-avoidance pairs) than the two stop-watch cues from different goal orientations (i.e., approach-avoidance pairs). For example, the approach cue for the 1-point condition had higher similarity in the striatum and vmPFC activity pattern to the approach cue for the 3-point condition than the avoidance cue for the same 1-point condition. These results may indicate that the two goal states are supported by distinct mechanisms in the reward network (Levorsen et al., 2021; Peelen & Downing, 2007). In addition, participants who showed more distinct activation patterns in the striatum across the approach and avoidance conditions reported more distinct subjective experiences between the two goal frame conditions. In contrast, the pattern of activity in the vmPFC was not significantly associated with subjective experiences. These results suggest that even when the level of striatal activation is similar, a pattern of activity in the striatum may be important to distinguish and support positive and negative motivational states.

Second, we observed differences between the two conditions in our whole brain analysis during the outcome phase. Specifically, there were some brain areas that responded similarly to the successful outcomes in the approach condition versus the failure outcomes in the avoidance condition. The majority of these are visual or motor related areas, indicating that these outcomes (i.e., success in approach condition and failure in avoidance condition) may have been preferentially attended to by participants. The strong activity observed in the visual area makes sense, because these two outcomes resulted in the visible change of the points to participants; therefore, they should have been salient to participants. One interesting result concerns the hippocampus. The hippocampus showed increased activity to failures than success in the avoidance condition but not in the approach condition. Previous research also suggests that the hippocampus is critical in dealing with an approach-avoidance conflict (i.e., when individuals approach stimuli that they rather want to avoid), although the implicated area often was more anterior than the cluster we observed (Bach et al., 2014; Loh et al., 2016; O'Neil et al., 2015). Relatedly, neurobiological theories of anxiety indicate that the hippocampus plays a critical role in anxiety (Gray & McNaughton, 1996), an emotion that individuals often experience when they focus on avoidance goals. Future studies should closely examine how the hippocampus helps in learning from failures particularly under avoidance goals.

In contrast to the hippocampus, we did not see any significant differences between the approach and the avoidance conditions in the reward network during the outcome phase. Behaviorally, the success feedback induced stronger happiness after the approach condition than the avoidance condition. This is consistent with the notion that a success in the approach condition leads to stronger happiness and enjoyment relative to a success in the avoidance condition (Elliot & Pekrun, 2007). Despite such differences in subjective experiences, the success feedback, relative to the failure feedback, evoked stronger activity in the striatum irrespective of the goal frames. These results are consistent with previous findings that the striatum was activated when an aversive event (e.g., pain) was not present and participants were relieved (Leknes et al., 2011; Navratilova et al., 2015). Thus, the lack of an aversive event (i.e., a loss of points) in the event of success in the avoidance condition can be still associated with striatum activity.

Another important observation is that the two goal frame conditions were not significantly different in other brain regions relevant to emotions (such as the amygdala and the insula). This is in contrast to previous studies on prevention and promotion focus; these studies revealed that individual differences in prevention versus promotion focus were associated with individual differences in amygdala activity to emotional stimuli, such as positive/negative concepts or positive/negative emotional images (Cunningham et al., 2005, 2010). However, in these studies, participants were typically presented with unique stimuli in each trial. In contrast, in the present study, the stopwatch was similar across trials, which may have induced the habituation of the amygdala and prevented us from seeing any effects of goals on amygdala activity (Weierich et al., 2010). In fact, other studies without emotional stimuli did not observe the effects of individual differences in goal orientation on amygdala activity (Belayachi et al., 2015; Hahn et al., 2009). Thus, the effects of goal orientation on amygdala activity may be stronger in situations where individuals face emotionally salient and distinct stimuli.

It is important to note several limitations of the present study. First, our sample size was modest. Even though we observed reliable differences between the two goal conditions in behavioral measures, the relatively small sample size may have led to the lack of power to detect the effects of goal frames in the univariate fMRI analysis. Relatedly, due to the modest sample size, our study was not well powered to investigate the effects of individual differences. Thus, caution is required when interpreting our results on individual differences in the pattern similarity index; future research needs to examine whether the results will be replicated in an independent and larger sample. In addition, previous research revealed that individual differences in their goal orientation play an important role in modulating the effects of situational goal frame manipulations (Cunningham et al., 2005; Touryan et al., 2007). Further research is required to understand the interaction between the approach versus avoidance goal manipulation and individual differences in the salience of these two goals.

Second, our goal frame manipulation was different from those used in previous behavioral or neuroimaging studies. In previous neuroimaging studies, the goal frame often was manipulated by using monetary rewards (as described in the Introduction) (Schlund et al., 2011); this means that achieving a goal has a clear personal benefit for participants (i.e., they would be able to earn more money). Previous studies also did not always control for performance (Elliot & Harackiewicz, 1996; Gee et al., 2018; Schlund et al., 2011). The manipulations we used were different from them; there were no extrinsic rewards associated with the goal, and performance was experimentally controlled so that there was no difference in performance across the two goal frame conditions. Nevertheless, we replicated previously observed effects of goal frames, such as increased enjoyment under the approach goal, increased anxiety under the avoidance goal, and increased engagement under the approach goal (Elliot & Harackiewicz, 1996; Gee et al., 2018; Higgins et al., 1997). This suggests that our manipulation was successful to alter the saliency of participants’ goals. At the same time, given that most behavioral measures were obtained after the scanning session, it is possible that their responses were biased by memory errors. Future research may want to assess subjective experiences without relying too much on post-hoc self-reports.

## Conclusions

The present study demonstrates both the similarity and the uniqueness in the role of the reward network in approach versus avoidance goals. As observed in previous psychological studies, we observed that the same task is perceived differently depending on whether it is framed as approach or avoidance goals. Despite the marked difference in subjective motivational states, the reward network showed similar levels of activity across the two conditions; such that the striatum, VTA, and SN showed higher levels of activity to task cues in the approach goal condition as well as the avoidance goal condition relative to the control cue. The RSA also revealed that the approach condition and the avoidance condition were represented similarly in the striatum and vmPFC relative to the control condition. However, RSA also indicates differences between the two goal frames, suggesting that the two goal frames were represented differently within the striatum and vmPFC. These results suggest that the reward network is involved both in general motivational engagement and in the emotional valence of subjective motivational experiences.

### Supplementary information

ESM 1 (DOCX 153 MB)
