# Supplementary material for: Motivated with joy or anxiety: Does approach-avoidance goal framing elicit differential reward-network activation in the brain?
Source: Cogn Affect Behav Neurosci. 2024 Jan 30;24(3):469–90. doi: 10.3758/s13415-024-01154-3 (PMC11078806; doi:10.3758/s13415-024-01154-3)

Table S1. Significant clusters during the cue presentation in whole brain analysis

|  |  |  |  |  |  | MNI |  |
| --- | --- | --- | --- | --- | --- | --- | --- |
| Contrast | Voxels | Area | Z stat | H | x | y | z |
| *Approach > Control* | 57095 | Occipital Pole | 6.62 | R | 16 | -92 | -6 |
|  |  | Lateral Occipital Cortex | 6.1 | R | 38 | -84 | -4 |
|  |  | Supramargical Gyrus | 6.1 | R | 38 | -44 | 40 |
|  |  | Supramargical Gyrus | 6.08 | R | 42 | -44 | 44 |
|  |  | Supramargical Gyrus | 6.08 | L | -42 | -42 | 38 |
|  |  | Supramargical Gyrus | 6.01 | L | -52 | -42 | 40 |
|  | 1480 | Frontal Pole | 5.2 | L | -44 | 38 | 24 |
|  |  | Frontal Pole | 4.74 | L | -36 | 42 | 18 |
|  |  | Middle Frontal Gyrus | 4.65 | L | -38 | 32 | 22 |
|  |  | Frontal Pole | 4.5 | L | -30 | 44 | 20 |
|  |  | Frontal Pole | 4.47 | L | -34 | 48 | 22 |
|  |  | Frontal Pole | 4.42 | L | -40 | 42 | 18 |
|  | 200 | Intracalcarine Cortex | 4.17 | R | 14 | -66 | 12 |
|  |  | Intracalcarine Cortex | 4.05 | R | 12 | -66 | 16 |
|  |  | Lingual Gyrus | 3.89 | R | 24 | -56 | 4 |
|  |  | Intracalcarine Cortex | 3.78 | R | 12 | -66 | 6 |
|  | 188 | Frontal Pole | 4.18 | R | 20 | 34 | -26 |
|  |  | Frontal Pole | 3.95 | R | 26 | 44 | -22 |
|  |  | Frontal Pole | 3.75 | R | 28 | 52 | -20 |
|  |  | Frontal Pole | 3.71 | R | 34 | 54 | -22 |
|  |  | Frontal Pole | 3.7 | R | 38 | 56 | -20 |
|  |  | Frontal Pole | 3.2 | R | 18 | 50 | -22 |
| *Avoidance > Control* | 61167 | Lateral Occipital Cortex | 6.42 | L | -40 | -78 | -4 |
|  |  | Occipital Pole | 6.39 | R | 26 | -90 | -12 |
|  |  | Temporal Occipital Fusiform Cortex | 6.34 | R | 38 | -50 | -26 |
|  |  | Supramargical Gyrus | 6.33 | L | -48 | -36 | 36 |
|  |  | Temporal Occipital Fusiform Cortex | 6.31 | L | -38 | -64 | -18 |
|  |  | Temporal Occipital Fusiform Cortex | 6.27 | R | 40 | -56 | -22 |
|  | 110 | Middle Temporal Gyrus | 4.35 | R | 52 | -22 | -10 |
|  | 109 | Frontal Pole | 4.31 | R | 26 | 38 | -24 |
|  |  | Frontal Pole | 3.72 | R | 16 | 50 | -22 |
|  |  | Frontal Pole | 3.39 | R | 20 | 42 | -18 |
| *Approach > Avoidance* | |  |  |  |  |  |  |
|  |  | **NS** |  |  |  |  |  |
| *Avoidance > Approach* | |  |  |  |  |  |  |
|  |  | **NS** |  |  |  |  |  |

Table S2. Significant clusters during the outcome phases in whole brain analysis from other contrasts.

|  |  |  |  |  |  | MNI |  |
| --- | --- | --- | --- | --- | --- | --- | --- |
| Contrast | Voxels | Area | Z stat | H | x | y | z |
| *Avoidance Success > Approach Success* | | |  |  |  |  |  |
|  | NS |  |  |  |  |  |  |
| *Approach Success > Avoidance Success* | | |  |  |  |  |  |
|  | 7187 | Lateral Occipital Cortex | 5.34 | R | 36 | -86 | -6 |
|  |  | Occipital Pole | 5.32 | R | 34 | -68 | -18 |
|  |  | Lateral Occipital Cortex | 5.11 | R | 30 | -92 | -16 |
|  |  | Inferior Temporal Gyrus | 5.08 | R | 48 | -70 | -2 |
|  |  | Occipital Fusiform Gyrus | 5.07 | R | 52 | -58 | -14 |
|  |  | Lateral Occipital Cortex | 5.05 | R | 28 | -78 | -18 |
|  | 3886 | Lateral Occipital Cortex | 5.19 | L | -46 | -62 | -8 |
|  |  | Lateral Occipital Cortex | 4.98 | L | -50 | -70 | -6 |
|  |  | Lateral Occipital Cortex | 4.93 | L | -44 | -66 | -14 |
|  |  | Lateral Occipital Cortex | 4.9 | L | -46 | -74 | -10 |
|  |  | Occipital Fusiform Gyrus | 4.84 | L | -40 | -68 | -14 |
|  |  | Occipital Fusiform Gyrus | 4.72 | L | -32 | -70 | -16 |
|  | 511 | Precentral Gyrus | 5.04 | R | 50 | 8 | 24 |
|  |  | Precentral Gyrus | 4.97 | R | 46 | 8 | 24 |
|  |  | Precentral Gyrus | 4.15 | R | 42 | 4 | 32 |
|  |  | Precentral Gyrus | 3.99 | R | 46 | 2 | 44 |
|  |  | Precentral Gyrus | 3.87 | R | 42 | 0 | 42 |
|  |  | Precentral Gyrus | 3.75 | R | 44 | 0 | 38 |
|  | 160 | Cerebellum | 4.07 | L | -8 | -76 | -42 |
|  |  | Cerebellum | 3.31 | R | 2 | -80 | -44 |
|  |  | Cerebellum | 3.26 | L | -18 | -70 | -40 |
|  | 123 | Cerebellum | 4.07 | L | -2 | -54 | -34 |
|  |  | Cerebellum | 3.76 | L | 0 | -64 | -34 |
|  |  | Cerebellum | 3.64 | R | 6 | -54 | -34 |
|  | 122 | Precentral Gyrus | 4.27 | L | -52 | 4 | 34 |
| *Avoidance Failure > Approach Failure* | | |  |  |  |  |  |
|  | 6077 | Occipital Fusiform Gyrus | 5.69 | R | 34 | -66 | -14 |
|  |  | Occipital Fusiform Gyrus | 5.63 | R | 36 | -70 | -16 |
|  |  | Temporal Occipital Fusiform Cortex | 5.39 | R | 40 | -58 | -18 |
|  |  | Temporal Occipital Fusiform Cortex | 5.19 | R | 34 | -50 | -18 |
|  |  | Temporal Occipital Fusiform Cortex | 5.16 | R | 44 | -50 | -18 |
|  |  | Lateral Occipital Cortex | 5.12 | R | 44 | -62 | -10 |
|  | 4616 | Lateral Occipital Cortex | 5.4 | L | -46 | -70 | -12 |
|  |  | Temporal Occipital Fusiform Cortex | 5.27 | L | -32 | -60 | -16 |
|  |  | Lateral Occipital Cortex | 5.15 | L | -52 | -66 | -6 |
|  |  | Occipital Fusiform Gyrus | 5.14 | L | -36 | -68 | -12 |
|  |  | Lateral Occipital Cortex | 5.14 | L | -40 | -80 | -10 |
|  |  | Lateral Occipital Cortex | 5.05 | L | -36 | -86 | 8 |
|  | 399 | Inferior Frontal Gyrus | 4.46 | R | 46 | 8 | 22 |
|  |  | Inferior Frontal Gyrus | 4.24 | R | 42 | 12 | 26 |
|  |  | Inferior Frontal Gyrus | 4.15 | R | 42 | 14 | 22 |
|  |  | Inferior Frontal Gyrus | 4.09 | R | 42 | 10 | 20 |
|  |  | Inferior Frontal Gyrus | 3.97 | R | 46 | 12 | 16 |
|  |  | Inferior Frontal Gyrus | 3.68 | R | 54 | 14 | 28 |
|  | 124 | Precentral Gyrus | 3.93 | L | -50 | 4 | 34 |
|  |  | Precentral Gyrus | 3.91 | L | -52 | 10 | 32 |

Table S3. Loading of the First Principal Component into the Self-Report Measures

|  | Loading |
| --- | --- |
| Enjoyment (on task) | 0.417197 |
| Anxiety (on task) | -0.34871 |
| Enjoyment to the cue | 0.442582 |
| Anxiety to the cue | -0.19373 |
| Disappoint to the cue | -0.3372 |
| Engagement to the cue | 0.325297 |
| Excitement to the cue | 0.364847 |
| Happiness after success | -0.02282 |
| Relief after success | -0.27578 |
| Anxiety after failure | -0.14004 |
| Disappoint after failure | -0.14871 |

Figure S1. The beta values in the nucleus accumbens to success than failure outcomes in the approach and avoidance condition.


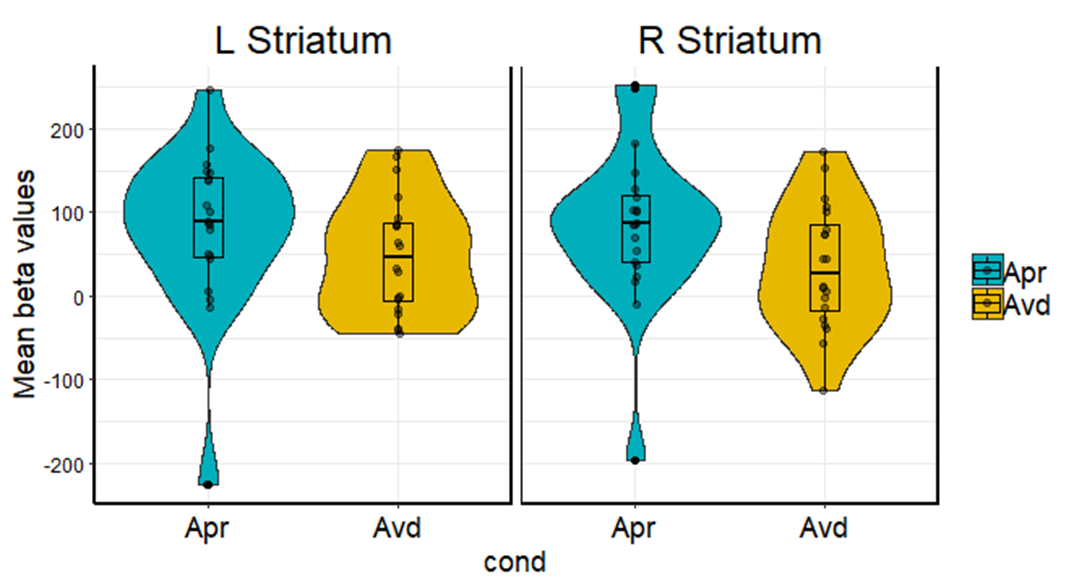


Figure S2. Significant clusters for the “approach cue > control cue” contrast (in red) and the “avoidance cue > control cue” contrast (in blue) in whole brain analysis. Areas shown in purple were activated in both of the contrast


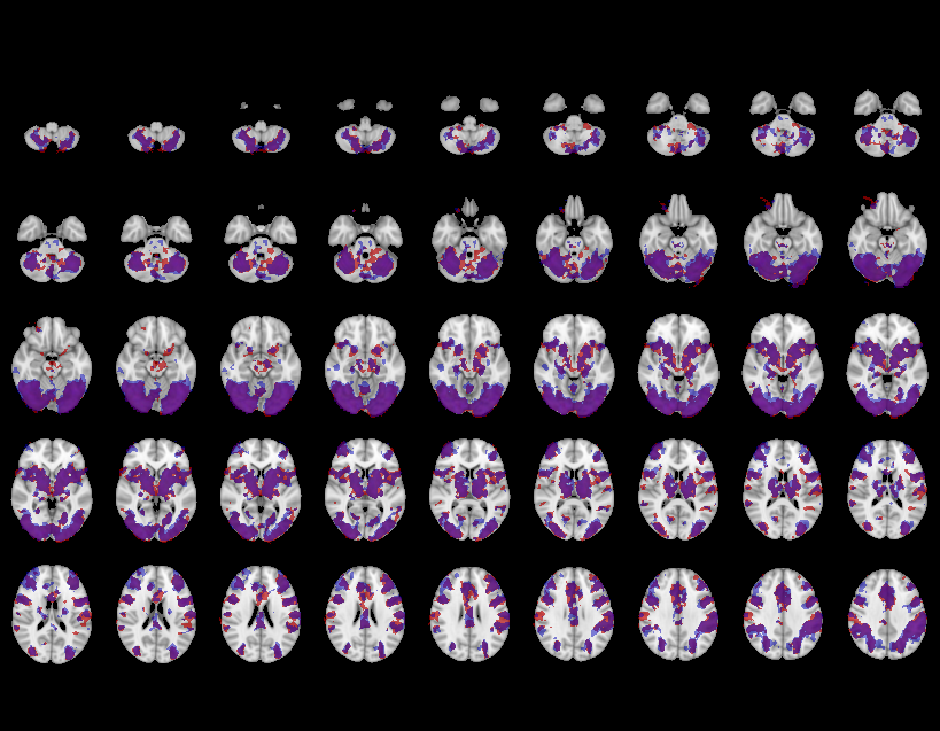


Figure S3. Significant clusters for “approach success > approach failure” contrast.


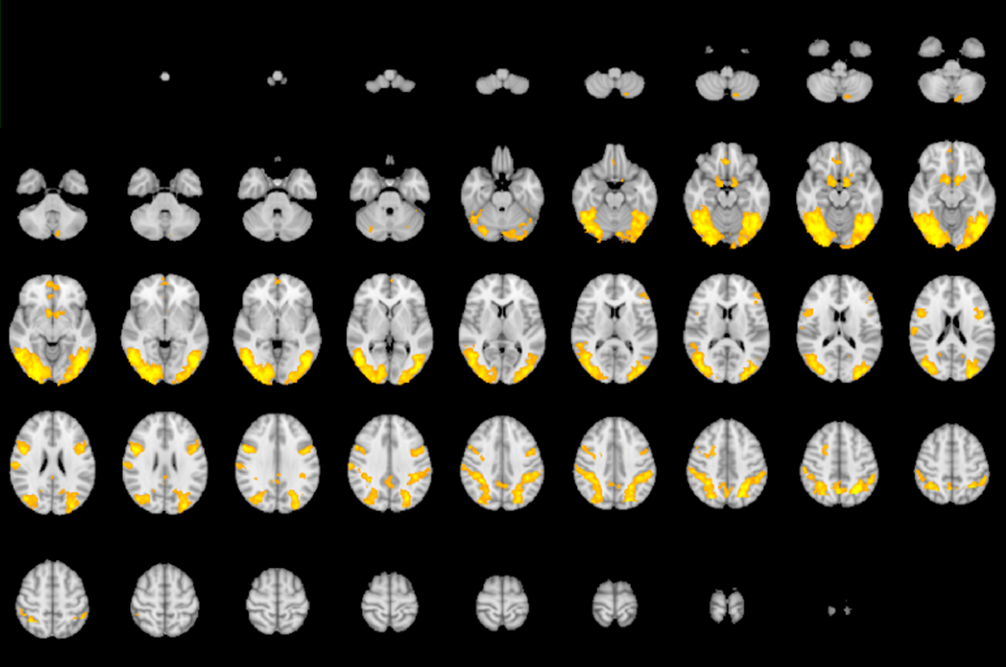


Figure S4. Significant clusters for “avoidance failure > avoidance success” contrast.


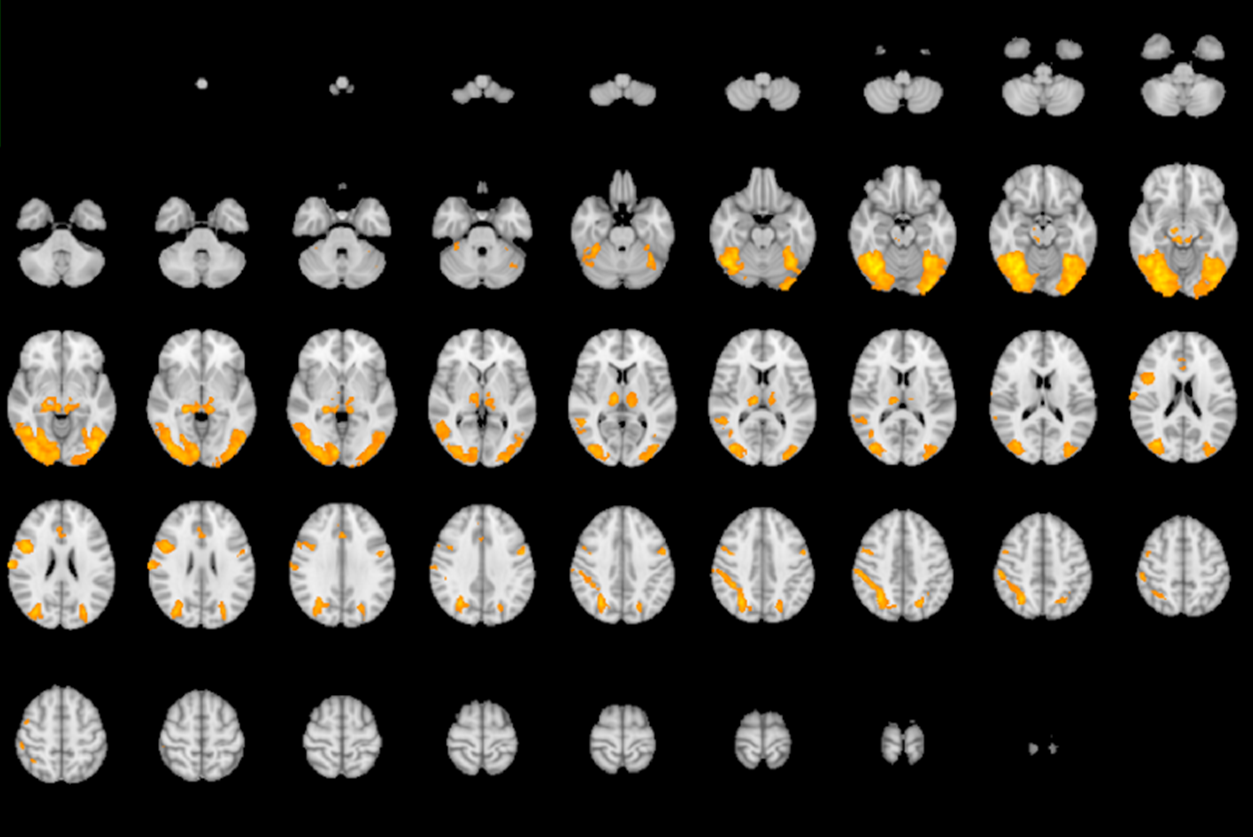

Supplement: Supplementary file 1 — (DOCX 153 MB) [file 13415_2024_1154_MOESM1_ESM.docx]
